# Fetal ultrasound parameters associated with N-terminal pro-B-type natriuretic peptide levels in umbilical cord blood: a cross-sectional study

**DOI:** 10.1186/s40748-026-00286-3

**Published:** 2026-08-12

**Authors:** Shoko Saito, Mari Tadakawa, Hirotaka Hamada, Noriyuki Iwama, Masatoshi Saito

**Affiliations:** 1 Department of Obstetrics and Gynecology, Tohoku University Graduate School of Medicine, Sendai, Miyagi 9808575 Japan; 2 Department of Obstetrics and Gynecology, Tohoku University Hospital, Sendai, Miyagi 9808574 Japan; 3 Center for Perinatal Medicine, Tohoku University Hospital, Sendai, Miyagi 9808574 Japan; 4 Department of Maternal and Fetal Therapeutics, Tohoku University Graduate School of Medicine, Sendai, Miyagi 9808575 Japan

**Keywords:** Heart failure, Fetal heart, Heart function tests, Ultrasonography, Prenatal, Doppler Ultrasonography, Hemodynamics, Natriuretic peptide, brain, Fetal blood

## Abstract

**Background:**

Current Doppler and functional ultrasound parameters, such as the cardiovascular profile score, umbilical artery pulsatility index, and ductus venosus pulsatility index, are valuable for detecting advanced fetal cardiac decompensation; however, they have limited ability in identifying subtle hemodynamic changes. This critical gap in current fetal assessment highlights the need for reliable markers that can identify subtle cardiac stress before overt failure. In this study, we aimed to identify fetal ultrasound parameters associated with umbilical venous N-terminal pro-B-type natriuretic peptide (NT-proBNP) levels, a biomarker associated with fetal cardiac load.

**Methods:**

We conducted a single-center, cross-sectional study at a tertiary perinatal medical center, recruiting women with singleton pregnancies undergoing cesarean delivery at ≥22 weeks of gestation. Fetal ultrasonographic examinations were performed within 7 days before delivery, and NT-proBNP levels were measured in umbilical venous blood collected immediately after birth. NT-proBNP values were natural log-transformed (Ln NT-proBNP) to address the non-normality of the residuals in the regression models, and gestational age-adjusted linear regression analyses were performed.

**Results:**

A total of 202 pregnant women were included in the analyses. A higher left to right cardiac output (LCO/RCO) ratio was significantly associated with lower Ln NT-proBNP levels (estimate: −0.206; 95% confidence interval [CI]: −0.314 to −0.097), whereas a higher anterior tibial artery to renal artery peak systolic velocity (ATA-PSV/RA-PSV) ratio was significantly associated with higher Ln NT-proBNP levels (estimate: 0.149, 95% CI: 0.047 to 0.251).

**Conclusions:**

LCO/RCO and ATA-PSV/RA-PSV were associated with NT-proBNP levels. LCO/RCO consistently improved model performance. While ATA-PSV/RA-PSV may reflect aspects of peripheral circulation not captured by conventional indices, its incremental value beyond gestational age was modest. Further studies are needed to validate these findings and clarify their clinical significance.

**Trial registration:**

This study was prospectively registered for transparency in the UMIN Clinical Trials Registry (UMIN-CTR; ID: UMIN000047081, registered on March 4, 2022), as it was an observational cross-sectional study without any intervention or randomization.

**Supplementary Information:**

The online version contains supplementary material available at 10.1186/s40748-026-00286-3.

## Background

Advances in ultrasonographic devices and imaging technology have enabled detailed evaluation of fetal cardiac function [1] and blood flow in small peripheral vessels [2]. These developments highlight the potential of ultrasonography as a noninvasive tool for detecting subtle signs of fetal cardiac stress [3, 4].

The transition at birth, from fetal to neonatal circulation, is a universal physiological process that demands precise hemodynamic adaptation [5]. This transition inevitably imposes a significant hemodynamic load on the fetal cardiovascular system, and adequate cardiac reserve is required to maintain circulatory stability. In cases of impaired cardiovascular transitions, an increased risk of circulatory failure and secondary complications such as hemorrhage and ischemia exists [6]. Moreover, in conditions such as fetal growth restriction (FGR), hypertensive disorders of pregnancy (HDP), and in late gestation with placental aging, increased vascular resistance in the umbilical-placental circulation may contribute to additional hemodynamic stress on the fetal heart [7].

In adults, natriuretic peptide (NP) levels increase before overt heart failure and are widely used for diagnosis and monitoring [8]. Among these, N-terminal pro-B-type NP (NT-proBNP) is preferred for its biochemical stability, longer half-life, and strong correlation with BNP levels [9].

Elevated umbilical cord blood NP levels, including NT-proBNP, have been linked to fetal and neonatal heart failure [10, 11], suggesting that NT-proBNP levels in umbilical blood could be monitored during pregnancy, enabling assessment of fetal cardiac function. However, prenatal sampling of umbilical blood is highly invasive for routine clinical care, making noninvasive surrogates essential.

Fetal ultrasound parameters offer a promising alternative. Conventional parameters, such as the cardiovascular profile (CVP) score, are valuable for identifying advanced fetal compromise; however, they generally reflect late-stage decompensated heart failure [12, 13] and are limited in identifying early adaptive responses, leaving a gap in fetal surveillance. This gap highlights the need for reliable, noninvasive markers capable of detecting subtle fetal hemodynamic stress before overt failure. In this study, we aimed to identify fetal ultrasound parameters associated with NT-proBNP levels in umbilical blood.

## Methods

### Study population

We conducted a single-center, cross-sectional study at a tertiary perinatal medical center. We recruited women with singleton pregnancies who underwent cesarean delivery at ≥22 weeks of gestation between April 2022 and March 2024, and met the following criteria: confirmed gestational age (GA) using first-trimester ultrasound (≤13 weeks of gestation), as both NT-proBNP levels and fetal ultrasonographic measurements are affected by GA, and absence of fetal chromosomal abnormalities. To avoid the potential effects of labor on fetal cardiac function and ensure optimal conditions for fetal ultrasonographic measurements, only cases of cesarean delivery were included. Cesarean deliveries were performed for standard obstetric indications, including both elective and emergency procedures.

### Ultrasonographic measurements

Fetal ultrasonographic measurements were performed using Voluson E10 (GE Healthcare, USA) or Aplio i800 (Canon Medical Systems, Japan). Examinations were conducted by experienced obstetricians with expertise in fetal cardiac function measurements. Measurements were performed only when the fetus was in a stable state, without (a) significant maternal or fetal movements, (b) fetal breathing-like movements, and (c) fetal heart rate abnormalities (<110 or > 160 beats per minute [bpm]). Doppler blood flow velocity measurements were obtained at an insonation angle of <60° with appropriate angle correction. Based on standard vascular ultrasound practice, an insonation angle of less than 60° is considered acceptable when appropriate angle correction is applied [14]. Efforts were made to keep the insonation angle as small as feasible to minimize measurement error. Ultrasonographic measurements obtained within 7 days before the cesarean section were included. In cases with multiple measurements within this time window, the measurement closest to delivery was used for analysis.

To minimize potential measurement bias, a predefined measurement protocol was applied based on previously established methods for each ultrasound parameter [15–24], and a consistent measurement approach was used across examiners. Stored ultrasonographic images were retrospectively reviewed by the lead investigator (S.S.) to confirm measurement accuracy, and only those that met the predefined measurement criteria were included in the analysis. Although standardization was attempted, inter- and intra-observer variability in ultrasonographic measurements was not formally assessed.

The ultrasonographic measurements were categorized into four domains: fetal growth, arterial Doppler, venous Doppler, and cardiac function. Fetal growth parameters included biparietal diameter (BPD), abdominal circumference (AC), femur length (FL), and estimated fetal weight (EFW). Arterial Doppler measurements included pulsatility index (PI) and peak systolic velocity (PSV) of the middle cerebral artery (MCA), descending aorta (Ao), renal artery (RA), and anterior tibial artery (ATA). Umbilical artery (UmbA) PI and qualitative assessment of end-diastolic flow were also evaluated. The cerebroplacental ratio (CPR) was calculated as MCA-PI/UmbA-PI. Venous Doppler assessments included qualitative evaluation of pulsation in the umbilical vein and a-wave abnormalities in the ductus venosus, as well as measurement of preload index (PLI) and DV-PI. Cardiac function parameters included total cardiac dimension (TCD), cardiothoracic area ratio (CTAR), mitral and tricuspid valve early-to-atrial peak velocity ratios (MV-E/A, TV-E/A), myocardial performance index (MPI), left and right ventricular ejection fraction (LV-EF, RV-EF), left and right ventricular fractional shortening (LV-FS, RV-FS), cardiac output (left, right, and combined: LCO, RCO, and CCO), as well as mitral and tricuspid annular plane systolic excursion, among others.

In addition to the individual parameters, derived ratios were calculated based on the hypothesis that they might better reflect fetal hemodynamic adaptation compared to individual absolute values. The LCO/RCO ratio was included based on previous studies reporting changes in ventricular output balance under fetal stress [25, 26]. Ratios of ATA/RA, ATA/Ao, and RA/Ao were calculated using both PI and PSV to assess relative differences in blood flow between peripheral and central circulations [2, 27, 28]. In addition, within-vessel ratios of PI to PSV were calculated to explore potential differences related to local vascular characteristics.

The CVP score was retrospectively calculated based on stored ultrasonographic data. Both quantitative parameters (CTAR, LV-FS and RV-FS) and qualitative findings (hydrops, venous doppler abnormalities, and valvular regurgitation) were used for CVP score assessment.

### Laboratory measurements

Umbilical cord blood samples were collected immediately after cord clamping during the cesarean section. Samples were promptly placed into Ethylenediaminetetraacetic acid disodium salt, 2-hydrate (EDTA-2Na) tubes, centrifuged at 4 °C, 3000 rpm, for 10 min, and analyzed within 1 h using a portable immunofluorescence analyzer PATHFAST (PHC Holdings Corporation, Japan) to measure NT-proBNP levels.

In this study, venous and arterial NT-proBNP concentrations showed a strong correlation (*Spearman’s* ρ = 0.961, *p* < 0.001), consistent with a previous study [29]. Considering this correlation and the greater availability of venous samples owing to easier collection, analyses were performed using umbilical venous blood.

### Statistical analyses

Continuous variables are presented as medians (interquartile ranges), whereas categorical variables are presented as numbers (percentages). As NT-proBNP levels were right-skewed, and Q-Q plots indicated non-normality of residuals, natural logarithm-transformed NT-proBNP (Ln NT-proBNP) was used as the dependent variable.

Given that NT-proBNP levels decline with advancing GA [30], Spearman’s rank correlation was used to examine the association between GA and NT-proBNP levels. A directed acyclic graph (DAG) was constructed using DAGitty.net (Additional file 1), indicating that GA was the only confounder that required adjustment. Although other clinical factors such as FGR and HDP are known to be associated with NT-proBNP levels, these variables were considered potential mediators rather than confounders based on the DAG, and were therefore not included in the primary adjustment model. Neonatal outcomes, including UmbA pH and Apgar score, were described as baseline characteristics but were not included as covariates because they occur after fetal ultrasonographic assessment.

Linear regression analyses were performed with adjustment for GA at the time of ultrasonographic measurement (Model 2). Unadjusted (Model 1), GA-adjusted (Model 2), and additionally adjusted models including clinical factors such as FGR and HDP (Model 3) are presented in Additional file 2 as part of sensitivity analyses. Each fetal ultrasound measurement was included in the model separately as an explanatory variable. Linear regression analyses were performed for all fetal ultrasound measurements using standardized continuous values (per 1 standard deviation [1 SD]) and reported regression coefficients (estimates) with 95% confidence intervals (95% CIs). We also categorized the ultrasound measurements into quartiles (Q1–Q4), treating Q1 as the reference group, and performed regression analyses accordingly. For CVP score, which is an ordinal variable indicating the cardiovascular status, a score of 10 was used as the reference category to reflect clinical interpretation. To assess the linear association between fetal ultrasound measurements and NT-proBNP levels, the *p*-value for the trend was calculated by modelling the quartiles of ultrasound measurements as continuous variables. Model performance was assessed using the Akaike Information Criterion (AIC), adjusted R-squared (adjusted R^2^), and F-tests for nested models.

Sensitivity analyses were performed to assess the robustness of the findings. First, additional analyses were conducted with further adjustment for clinical factors, including FGR and HDP. Second, the study population was restricted to pregnancies at ≥ 34 and ≥37 weeks of gestation to evaluate associations independent of GA, and analyses were also restricted to cases with ultrasonographic measurements obtained within 1 day before delivery, considering the dynamic nature of both ultrasonographic measurements and NT-proBNP levels in umbilical cord blood. Furthermore, neonates were categorized based on the requirement for postnatal cardiovascular support, and NT-proBNP levels were compared between groups as an exploratory analysis. Cardiovascular support was defined as the administration of intravenous fluids or medications for circulatory stabilization.

Analyses were conducted using complete case data, excluding cases with missing values on a per-analysis basis without imputation. As the number of cases varied by analysis, the corresponding sample size (N) was reported individually. Statistical significance was defined as a two-sided *p*-value of <0.05. All statistical analyses were performed using the R software version 4.4.2 (R Core Team. R: A Language and Environment for Statistical Computing. Austria: R Foundation for Statistical Computing; 2024.)

## Results

### Characteristics of the study population

Among the 275 women who provided informed consent, 71 were excluded from the analysis owing to the absence of fetal ultrasonography within 7 days before delivery or lack of cord venous blood collection at cesarean section, and two participants were excluded owing to improper sample processing. A total of 202 women were included in the final analysis. Cesarean deliveries were performed for standard obstetrics indications, including both elective and emergency procedures. Among the included cases, 184 (91.1%) underwent elective cesarean delivery and 18 (8.9%) underwent emergency cesarean delivery. Among emergency cesarean deliveries, fetal indications accounted for 11 cases, whereas maternal indications accounted for 7. The baseline maternal and neonatal characteristics are summarized in Table 1.

**Table 1:** Baseline characteristics of the study population (*n* = 202)

Characteristic	Value
Maternal age (years)	35.0 (31.0–38.0)
<35 years	100 (49.5%)
≥35 years	102 (50.5%)
GA at delivery (weeks)	37.9 (37.5–38.3)
<37 weeks	18 (8.9%)
Cesarean delivery	
Elective	184 (91.1%)
Emergency	18 (8.9%)
Birthweight (g)	2836 (2624–3048)
<2500 g	38 (18.8%)
Male sex	98 (48.5%)
FGR	15 (7.4%)
HDP	14 (6.9%)
UA pH < 7.10	2 (1.0%)
Apgar score at 1 min < 7	26 (12.9%)
Apgar score at 5 min < 7	7 (3.5%)
NT-proBNP (pg/mL)	604.5 (443.2–939.5)

No missing data were noted for the variables in this table. Values are shown as median (Q1–Q3) or n (%). Gestational age at delivery corresponds to the timing of NT-proBNP blood sampling. FGR, HDP, UA pH < 7.10, and Apgar score at 1 minute < 7 have been reported to correlate with umbilical blood natriuretic peptide levels. None of the study cases had congenital heart disease, fetal arrhythmia, or required postnatal catecholamine treatment

Abbreviations: FGR, fetal growth restriction; GA, gestational age; HDP, hypertensive disorders of pregnancy; NT-proBNP, N-terminal pro-B-type natriuretic peptide; UA, umbilical artery

Clinical factors previously reported to be associated with NP levels in umbilical blood, such as FGR, HDP, UmbA pH < 7.10, and Apgar score at 1 min < 7 [7, 11, 31, 32] are also described. Median maternal age was 35.0 years (31.0–38.0 years), median GA at delivery was 37.9 weeks (37.5–38.3 weeks), and median birthweight was 2836 g (2624–3048 g). The rates of FGR, HDP, UmbA pH < 7.10, and Apgar score < 7 at 1 min were 7.4%, 6.9%, 1.0%, and 12.9%, respectively. No cases of fetal congenital heart disease, fetal arrhythmia, postnatal catecholamine use, or other major neonatal complications were identified. An exploratory comparison revealed higher NT-proBNP levels in neonates requiring postnatal cardiovascular support (*n* = 15) compared with those not requiring support (*n* = 187) (median 2478.0 pg/mL [1320.0–3887.5] vs 584.0 pg/mL [434.5–837.5]). However, the number of cases requiring support was limited, precluding definitive statistical interpretation. No cases required catecholamine support.

### GA at blood sampling and NT-proBNP levels

The median NT-proBNP level in umbilical venous blood was 604.5 pg/mL (443.3–939.5 pg/mL), showing a right-skewed, non-normal distribution (Additional file 3a). A moderate negative correlation was observed between GA at blood sampling and NT-proBNP levels (Spearman’s ρ = −0.408, *p* < 0.001) (Additional file 3b). We used the natural log-transformed variable (Ln NT-proBNP) as the outcome in subsequent analyses because the residuals of the raw NT-proBNP level models showed non-normality. The distribution of Ln NT-proBNP levels and its association with GA are shown in Additional file 4. The log-transformed distribution showed a substantial improvement in normality, although a mild right skew remained. The negative correlation with GA was preserved.

### Fetal ultrasonographic parameters associated with Ln NT-proBNP levels

**Fig. 1:**
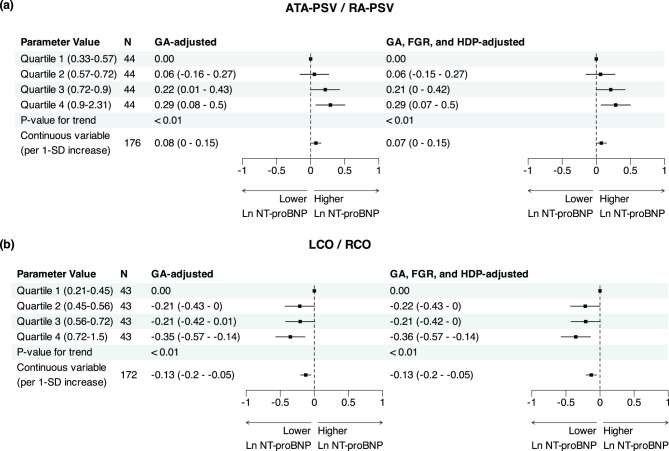
Associations of LCO/RCO (**a**) and ATA-PSV/RA-PSV (**b**) With Ln NT-proBNP. *1 SD = 0.22 LCO/RCO and 0.30 for ATA-PSV/RA-PSV. Abbreviations: ATA-PSV, anterior tibial artery peak systolic velocity; CI, confidence interval; FGR, fetal growth restriction; GA, gestational age; HDP, hypertensive disorders of pregnancy; LCO, left cardiac output; Ln NT-proBNP, natural logarithm of N-terminal pro B-type natriuretic peptide; RA-PSV, renal artery peak systolic velocity; RCO, right cardiac output; SD, standard deviation

Most measurements (93.6%) were taken within 1 day of delivery. Additional file 2 includes the associations between ultrasound parameters and Ln NT-proBNP in both the unadjusted (Model 1), GA-adjusted (Model 2), and additionally adjusted models including FGR and HDP (Model 3). Several parameters, such as EFW, UmbA-PI, CPR, and PLI, were significantly associated with Ln NT-proBNP in Model 1, but these associations were no longer significant in Model 2. In contrast, CTAR, LCO, LCO/RCO, and the ratio of ATA-PSV to RA-PSV (ATA-PSV/RA-PSV) remained significantly associated with Ln NT-proBNP in Model 2. However, the 1-SD change in LCO was deemed too large for clinical interpretation, and CTAR showed no clear dose–response relationship across quartiles. Therefore, LCO/RCO and ATA-PSV/RA-PSV were selected for presentation in the main results (Fig. 1).

**Fig. 2:**
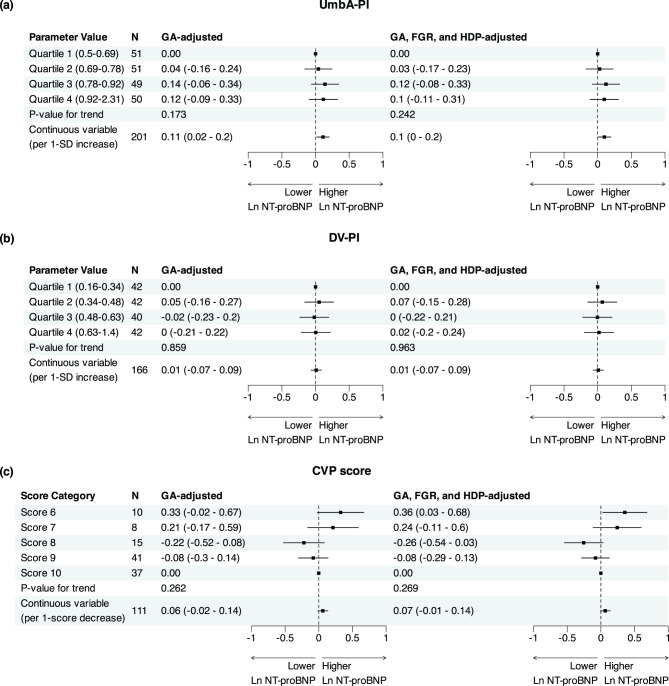
Association of UmbA-PI (**a**), DV-PI (**b**), and CVP score (**c**) With Ln NT-proBNP. *1 SD = 0.24 for UmbA PI and 0.21 for DV PI. Abbreviations: CI, confidence interval; CVP, cardiovascular profile; DV-PI, ductus venosus pulsatility index; FGR, fetal growth restriction; GA, gestational age; HDP, hypertensive disorders of pregnancy; Ln NT-proBNP, natural logarithm of N-terminal pro B-type natriuretic peptide; SD, standard deviation; UmbA-PI, umbilical artery pulsatility index

Associations between fetal ultrasonographic parameters and Ln NT-proBNP levels based on the GA-adjusted model are shown in Figs. 1 and 2, with additional adjustment for FGR and HDP also presented. Among previously established parameters reported to be associated with fetal heart failure or cord blood NT-proBNP levels, such as UmbA-PI, DV-PI, and CVP score (Fig. 2), only UmbA-PI showed a statistically significant positive association with Ln NT-proBNP per 1-SD increase, although the trend was not statistically significant (*p* = 0.173). No statistically significant associations were observed for the other parameters.

As shown in Fig. 1, LCO/RCO and ATA-PSV/RA-PSV were significantly associated with Ln NT-proBNP levels. Higher LCO/RCO was significantly associated with lower Ln NT-proBNP levels. In the 1-SD standardized continuous analysis, the estimate was −0.206 (95% CI: −0.314 to −0.097), and in the quartile-based model, Ln NT-proBNP levels were lower in higher quartiles (Q2–Q4) compared with those in Q1. The *p*-value for the trend was also statistically significant, indicating a linearly graded relationship (*p* < 0.01).

Conversely, higher ATA-PSV/RA-PSV was significantly associated with higher Ln NT-proBNP levels. In the 1-SD model, the estimate was 0.149 (95% CI: 0.047 to 0.251). In the quartile-based model, Ln NT-proBNP levels were higher in Q2–Q4 compared with those in Q1, with a significant *p*-value for trend supporting a linear graded relationship (*p* < 0.01).

These associations remained largely unchanged after additional adjustment for FGR and HDP. Sensitivity analyses restricted to pregnancies at ≥ 34 and ≥37 weeks of gestation, as well as analyses restricted to cases with ultrasonographic measurements obtained within 1 day before delivery, yielded similar results (Additional file 5).

### Model performance

The model comparison results are presented in Table 2. Compared with the model with GA alone, the addition of the ATA-PSV/RA-PSV only slightly improved the model performance, whereas the inclusion of LCO/RCO significantly enhanced the model’s fit. Models that included the CVP score had a smaller sample size (*N* = 86) as this variable had many missing values. Therefore, Table 2 focuses on the models with a larger sample size (*N* = 153). Additional file 6 shows additional model comparisons, including the CVP score. Notably, the addition of previously reported parameters (UmbA-PI, DV-PI, and CVP score) did not lead to a significant improvement in model performance.

**Table 2:** Model performance comparison for Ln NT-proBNP with ultrasound parameters added to a baseline model (*N* = 153)

Models	AIC	Adjusted R^2^	F-value	*p*-value
Model N Base model (no ultrasound parameter)	225.7298	0.411	–	–
Model A + LCO/RCO ratio (per 1-SD increase)*	215.6577	0.452	12.31	<0.001
Model B + ATA-PSV/RA-PSV ratio (per 1-SD increase)*	223.1974	0.424	4.51	0.035

*1 SD = 0.22 for LCO/RCO and 0.30 for ATA-PSV/RA-PSV

All F-values and *p*-values represent comparisons with Model N using ANOVA F-tests

Model N included only gestational age at the time of ultrasound examination

Model A included LCO/RCO in addition to Model N

Model B included ATA-PSV/RA-PSV in addition to Model N

Abbreviations: AIC, Akaike Information Criterion; ATA-PSV, anterior tibial artery peak systolic velocity; LCO, left cardiac output; RA-PSV, renal artery peak systolic velocity; RCO, right cardiac output; 1 SD, 1 standard deviation

Sensitivity analyses restricted to pregnancies at ≥ 34 and ≥37 weeks of gestation, as well as analyses restricted to cases with ultrasonographic measurements obtained within 1 day before delivery, showed generally consistent patterns, with LCO/RCO demonstrating robust improvement in model fit, whereas the contribution of ATA-PSV/RA-PSV varied across subgroups (Additional file 7).

## Discussion

In this study, we identified two parameters, LCO/RCO and ATA-PSV/RA-PSV, that were independently associated with cord blood NT-proBNP levels, even after adjusting for GA. However, their contributions to model performance were not consistent across parameters.

The novelty of this study lies in our focus on subclinical variations in fetal cardiac load, as reflected by continuous changes in NT-proBNP levels, rather than relying on overt heart failure or predefined diagnostic thresholds. These parameters can be derived from standard Doppler measurements and, therefore, can be integrated into routine obstetric ultrasound without additional procedural risk.

Conventional indices, including UmbA, DV, and CVP score, have been associated with fetal cardiac failure, fetal compromise, and adverse neonatal outcomes [12, 33, 34]. In contrast, we incorporated peripheral arterial circulation, which has been less extensively investigated in this context. Peripheral vascular resistance is a key determinant of cardiac load in the fetal circulation, where direct measurement of blood pressure is not feasible, and may provide complementary information to conventional indices. Doppler changes in peripheral and renal arteries can be observed in fetuses with FGR; however, their physiological significance and clinical utility remain incompletely understood [4, 35]. Although previous studies have been primarily focused on advanced pathological conditions [12, 13], the present findings suggest that meaningful hemodynamic information may also be captured within non-extreme ranges of NT-proBNP.

BNP, a cardiac hormone secreted in response to volume and pressure overload [36], reduces cardiac stress through vasodilation, diuresis, and suppression of sympathetic activity [37]. Upon release, proBNP is cleaved into biologically active BNP and inactive NT-proBNP, a stable and long-lasting marker of cardiac burden in circulation [9].

The fetal heart responds to increasing hemodynamic load through adaptive mechanisms that may progress to cardiac dysfunction and ultimately heart failure [13, 38]. Within this framework, early responses are characterized by activation of the sympathetic nervous system and renin-angiotensin-aldosterone system [39, 40]. Based on this physiological context, the parameters identified in this study may reflect subtle alterations in cardiac load within the continuum of hemodynamic adaptation, rather than overt cardiac dysfunction.

These responses lead to increased peripheral vascular resistance, and the elevated ATA-PSV and RA-PSV observed in our study may be consistent with this mechanism. In addition, sympathetic activation enhances myocardial contractility to maintain cardiac output [41]. However, the fetal left ventricle exhibits lower contractility than the right ventricle throughout gestation [1], which may limit its ability to adapt to increased afterload. This may contribute to the observed association between lower LCO/RCO and higher NT-proBNP levels. A previous clinical study reported that fetuses requiring emergency cesarean section or instrumental delivery owing to intrapartum fetal compromise showed decreased LCO, increased RCO, and a consequent reduction in the LCO/RCO ratio [25], which is consistent with our findings. In contrast, other studies have reported that under fetal stress, LCO increased while RCO decreased, suggesting brain-sparing redistribution of blood flow [42, 43]. This discrepancy may reflect differences in the underlying mechanisms of fetal stress responses, such as afterload-related ventricular dysfunction, as opposed to hypoxia-induced redistribution.

Elevated NT-proBNP levels have been linked with advanced fetal compromise, including cases of congenital heart disease, arrhythmia, and severe FGR [10, 26, 44, 45]. Our results extend this evidence by demonstrating associations in a population not limited to overt fetal cardiac failure, and by identifying indices that may be sensitive to subtle variations in fetal cardiac load. However, the clinical significance of modest elevations in NT-proBNP remains uncertain and warrants further investigation.

Compared with a model including GA alone, the addition of LCO/RCO consistently improved model performance, whereas the effect of ATA-PSV/RA-PSV was variable, suggesting that ATA-PSV/RA-PSV should be considered an exploratory finding requiring further investigation.

In this study, the population was derived from a tertiary perinatal center, where a wide range of clinical conditions are encountered in routine practice. Although this heterogeneity may be a limitation, additional subgroup and sensitivity analyses–including adjustment for FGR and HDP, restriction to cases with ultrasonographic measurements obtained within 1 day before delivery, and restriction to pregnancies delivered at ≥ 34 and ≥37 weeks of gestation – yielded consistent results, supporting the robustness of the findings. These findings suggest that the observed associations are unlikely to be solely explained by sample heterogeneity or measurement timing.

However, this study has some limitations. First, the clinical significance of NT-proBNP levels outside clearly elevated ranges remains uncertain. Second, the underlying physiological mechanisms, including the role of neurohumoral systems such as the renin-angiotensin-aldosterone system, were not directly assessed. Third, formal inter- and intra-observer reproducibility analyses were not performed, and external validation is required.

## Conclusion

LCO/RCO and ATA-PSV/RA-PSV were associated with umbilical venous NT-proBNP levels. LCO/RCO consistently improved model performance, whereas the incremental value of ATA-PSV/RA-PSV beyond gestational age was modest. These findings suggest that selected fetal ultrasonographic parameters may reflect aspects of fetal hemodynamic adaptation associated with NT-proBNP levels. However, the clinical significance of these associations remains uncertain. Further studies are needed to validate these findings and clarify their clinical significance.

## Electronic supplementary material

Below is the link to the electronic supplementary material.

Supplementary Material 1

Supplementary Material 2

Supplementary Material 3

Supplementary Material 4

Supplementary Material 5

Supplementary Material 6

Supplementary Material 7

Supplementary Material 8

## Data Availability

The datasets generated and/or analyzed during the current study are not publicly available due to ethical restrictions, but are available from the corresponding author on reasonable request.

## Abbreviations

AC: Abdominal circumference
AIC: Akaike Information Criterion
Ao: Descending aorta
ATA-PSV: Anterior tibial artery peak systolic velocity
ATA-PSV/RA-PSV: Ratio of anterior tibial artery peak systolic velocity to renal artery peak systolic velocity
BPD: Biparietal diameter
CCO: Combined cardiac output
CI: Confidence interval
CPR: Cerebroplacental ratio
CTAR: Cardiothoracic area ratio
CVP score: Cardiovascular profile score
DAG: Directed acyclic graph
DV: Ductus venosus
EF: Ejection fraction
EFW: Estimated fetal weight
FGR: Fetal growth restriction
FL: Femur length
HDP: Hypertensive disorders of pregnancy
LCO: Left cardiac output
LCO/RCO: Ratio of left to right cardiac output
Ln NT-proBNP: Natural log-transformed N-terminal pro-B-type natriuretic peptide
MCA: Middle cerebral artery
MPI: Myocardial performance index
MV: Mitral valve
NT-proBNP: N-terminal pro-B-type natriuretic peptide
PI: Pulsatility index
PLI: Preload index
PSV: Peak systolic velocity
RA-PSV: Renal artery peak systolic velocity
RCO: Right cardiac output
SD: Standard deviation
TCD: Total cardiac dimension
TV: Tricuspid valve
UmbA: Umbilical artery
