# Supplementary material for: Fetal ultrasound parameters associated with N-terminal pro-B-type natriuretic peptide levels in umbilical cord blood: a cross-sectional study
Source: Matern Health Neonatol Perinatol. 2026 Aug 12;12:39. doi: 10.1186/s40748-026-00286-3 (PMC13463724; doi:10.1186/s40748-026-00286-3)
Supplement: Supplementary file 1 [file 40748_2026_286_MOESM1_ESM.pdf]

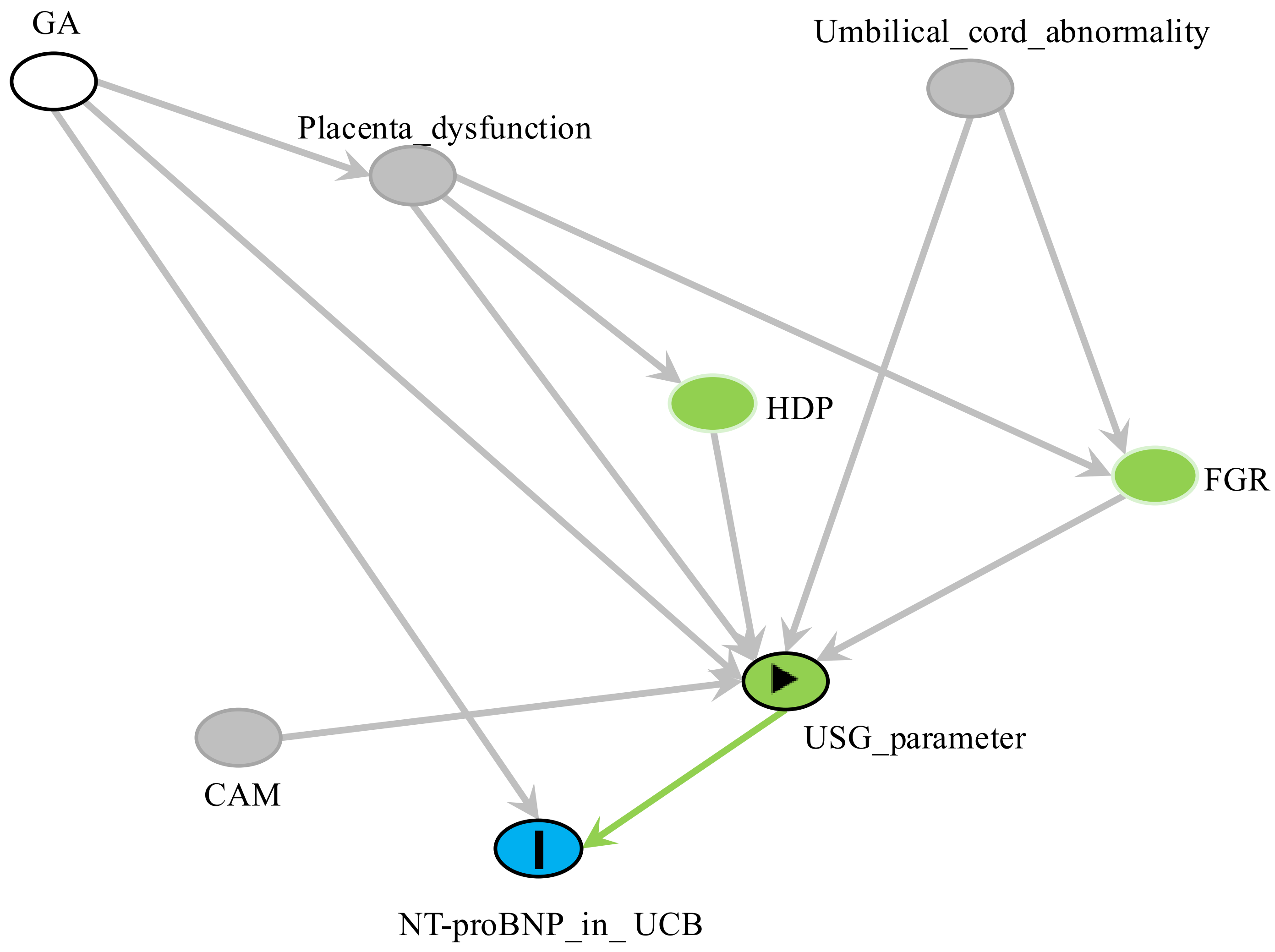
GA
Umbilical_cord_abnormality
Placenta_dysfunction
HDP
FGR
USG_parameter
CAM
NT-proBNP_in_ UCB
