# Supplementary material for: Fetal ultrasound parameters associated with N-terminal pro-B-type natriuretic peptide levels in umbilical cord blood: a cross-sectional study
Source: Matern Health Neonatol Perinatol. 2026 Aug 12;12:39. doi: 10.1186/s40748-026-00286-3 (PMC13463724; doi:10.1186/s40748-026-00286-3)
Supplement: Supplementary file 2 [file 40748_2026_286_MOESM3_ESM.pdf]

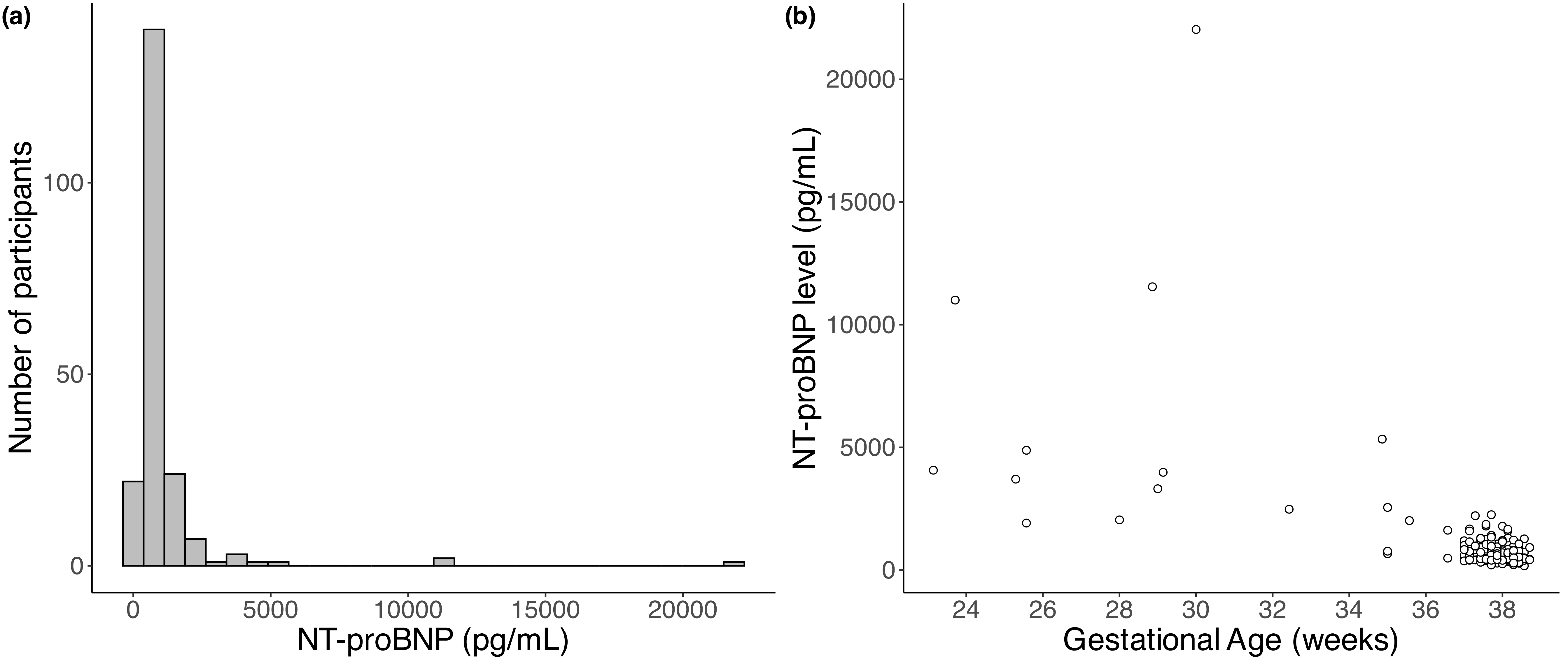
(a)
Number of participants
0
50
100
0
5000
10000
15000
20000
NT-proBNP (pg/mL)
(b)
NT-proBNP level (pg/mL)
0
5000
10000
15000
20000
24
26
28
30
32
34
36
38
Gestational Age (weeks)
