# Supplementary material for: Fetal ultrasound parameters associated with N-terminal pro-B-type natriuretic peptide levels in umbilical cord blood: a cross-sectional study
Source: Matern Health Neonatol Perinatol. 2026 Aug 12;12:39. doi: 10.1186/s40748-026-00286-3 (PMC13463724; doi:10.1186/s40748-026-00286-3)
Supplement: Supplementary file 3 [file 40748_2026_286_MOESM4_ESM.pdf]

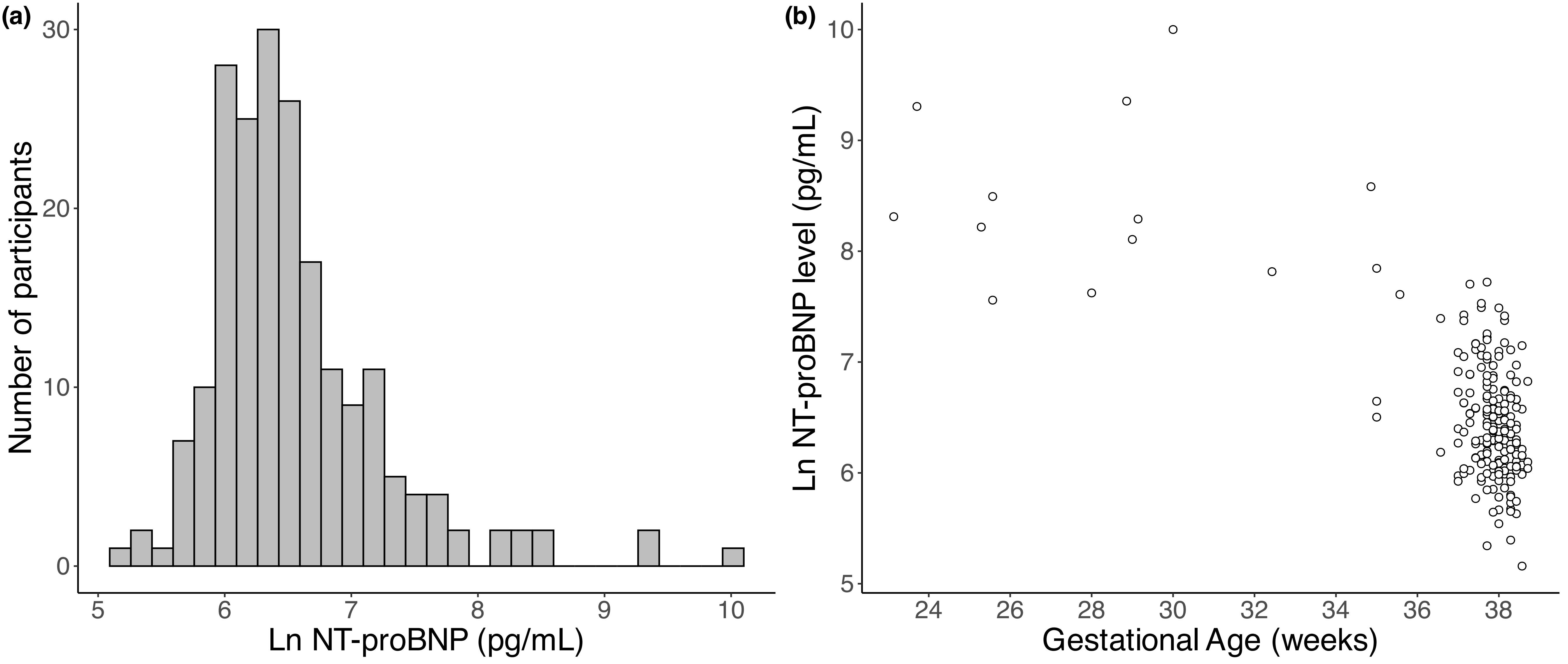

(a)
Number of participants
0
10
20
30
5
6
7
8
9
10
Ln NT-proBNP (pg/mL)
(b)
Ln NT-proBNP level (pg/mL)
5
6
7
8
9
10
24
26
28
30
32
34
36
38
Gestational Age (weeks)
