# Supplementary material for: Fetal ultrasound parameters associated with N-terminal pro-B-type natriuretic peptide levels in umbilical cord blood: a cross-sectional study
Source: Matern Health Neonatol Perinatol. 2026 Aug 12;12:39. doi: 10.1186/s40748-026-00286-3 (PMC13463724; doi:10.1186/s40748-026-00286-3)
Supplement: Supplementary file 4 [file 40748_2026_286_MOESM8_ESM.docx]

**ADDITIONAL FILES**

**Additional file 1**

**File format:** .pdf

**Title of data:** Figure S1. Directed acyclic graph (DAG)

**Description of data:** In the DAG generated using DAGitty.net, the green node with a triangle indicates the exposure variable, and the blue node indicates the outcome variable. The variables outlined in black represents the adjusted variable, whereas green nodes without a triangle indicate ancestors of the exposure that were not selected for adjustment.

Abbreviations: CAM, chorioamnionitis; FGR, fetal growth restriction; GA, gestational age; HDP, hypertensive disorder of pregnancy; NT-proBNP, N-terminal pro-B-type natriuretic peptide; UCB, umbilical cord blood; USG, ultrasonographic

**Additional file 2**

**File format:** .xlsx

**Title of data:** Table S1. Association of ultrasound parameters with Ln NT-proBNP

**Description of data:** 1-SD continuous and trend-based models are shown. Quartile-based results and cut-off ranges are not included due to space limitations. Model 1: Unadjusted model. Model 2: Adjusted for GA at ultrasound examination. Model 3: Adjusted for GA, FGR, and HDP.

Abbreviations: AC, abdominal circumference; Ao, aorta; ATA, anterior tibial artery; BPD, biparietal diameter; CCO, combined cardiac output; CCOkg, combined cardiac output per kg; CI, confidence interval; CPR, cerebroplacental ratio; CTAR, cardiothoracic area ratio; DV, ductus venosus; EA, E/A ratio (early and atrial waves); EFW, estimated fetal weight; ET, ejection time; FGR, fetal growth restriction; FL, femur length; GA, gestational age; HDP, hypertensive disorder of pregnancy; ICT, isovolumetric contraction time; IRT, isovolumetric relaxation time; LCO, left cardiac output; LVEF, left ventricular ejection fraction; LVFS, left ventricular fractional shortening; MAPSE, mitral annular plane systolic excursion; MCA, middle cerebral artery; MPI, myocardial performance index; MV, mitral valve; N, number of participants; PI, pulsatility index; PLI, pre-load index; PSV, peak systolic velocity; RA, renal artery; RCO, right cardiac output; RVEF, right ventricular ejection fraction; RVFS, right ventricular fractional shortening; SD, standard deviation; TAPSE, tricuspid annular plane systolic excursion; TCD, total cardiac dimension; TV, tricuspid valve; UmbA, umbilical artery; aS, a/S ratio.

**Additional file 3**

**File format:** .pdf

**Title of data:** Figure S2. Distribution of umbilical venous NT-proBNP levels

**Description of data:** (a) Histogram of NT-proBNP levels in umbilical venous blood. (b) Scatter plot of gestational age at blood sampling and NT-proBNP levels (Spearman’s *ρ* = -0.408, P < 0.001).

Abbreviations: NT-proBNP, N-terminal pro B-type natriuretic peptide.

**Additional file 4**

**File format:** .pdf

**Title of data:** Figure S3. Distribution of umbilical venous Ln NT-proBNP levels

**Description of data:** (a) Histogram of Ln NT-proBNP levels in umbilical venous blood.

(b) Scatter plot of gestational age at blood sampling and Ln NT-proBNP levels (Spearman’s *ρ* = -0.408, P < 0.001).

Abbreviations: Ln, natural logarithm; NT-proBNP, N-terminal pro B-type natriuretic peptide.

**Additional file 5**

**File format:** .xlsx

**Title of data:** Table S2. Sensitivity analyses of selected ultrasonographic parameters associated with Ln NT-proBNP

**Description of data:** Estimates are shown per 1-SD increase, except for CVP score, which is presented per 1-score decrease. Analyses include the main dataset and sensitivity analyses restricted to cases with ultrasonographic measurements obtained within 1 day before delivery and to pregnancies delivered at ≥34 and ≥37 weeks of gestation.

Abbreviations: ATA-PSV, anterior tibial artery peak systolic velocity; CVP score, cardiovascular profile score; DV-PI, ductus venosus pulsatility index; GA, gestational age; LCO, left cardiac output; RA-PSV, renal artery peak systolic velocity; RCO, right cardiac output; UmbA-PI, umbilical artery pulsatility index.

**Additional file 6**

**File format:** .xlsx

**Title of data:** Table S3. Model performance comparison for Ln NT-proBNP with ultrasound parameters added to a baseline model (N = 86)

**Description of data:** * 1-SD = 0.22 for LCO/RCO ratio, 0.30 for ATA-PSV/RA-PSV ratio, 0.56 for UmbA-PI, and 0.10 for DV-PI. Model N included only gestational age at the time of ultrasound examination. Model A included LCO/RCO ratio in addition to Model N. Model B included ATA-PSV/RA-PSV ratio in addition to Model N. Model C included UmbA-PI in addition to Model N. Model D included DV-PI in addition to Model N. Model E included CVP score in addition to Model N.

Abbreviations: ATA-PSV, anterior tibial artery peak systolic velocity; CVP score, cardiovascular profile score; DV-PI, ductus venosus pulsatility index; LCO, left cardiac output; RA-PSV, renal artery peak systolic velocity; RCO, right cardiac output; UmbA-PI, umbilical artery pulsatility index.

**Additional file 7**

**File format:** .xlsx

**Title of data:** Table S4. Sensitivity analyses of model performance for selected ultrasonographic parameters added to the baseline model

**Description of data:** * 1 SD = 0.22 for LCO/RCO and 0.30 for ATA-PSV/RA-PSV

Sensitivity analyses restricted to cases with ultrasonographic measurements obtained within 1 day before delivery and to pregnancies delivered at ≥34 and ≥37 weeks of gestation. Model N included only gestational age at the time of ultrasound examination. Model A included LCO/RCO in addition to Model N. Model B included ATA-PSV/RA-PSV in addition to Model N. All F-values and P-values represent comparisons with Model N using ANOVA F-tests.

AIC, Akaike Information Criterion; ATA-PSV, anterior tibial artery peak systolic velocity; GA, gestational age; LCO, left cardiac output; RA-PSV, renal artery peak systolic velocity; RCO, right cardiac output; 1 SD, 1 standard deviation.
